# Nanocontainers made of Various Materials with Tunable Shape and Size

**DOI:** 10.1038/srep02238

**Published:** 2013-07-19

**Authors:** Xianglong Zhao, Guowen Meng, Fangming Han, Xiangdong Li, Bensong Chen, Qiaoling Xu, Xiaoguang Zhu, Zhaoqin Chu, Mingguang Kong, Qing Huang

**Affiliations:** 1 Key Laboratory of Materials Physics, and Anhui Key Laboratory of Nanomaterials and Nanostructures, Institute of Solid State Physics, Chinese Academy of Sciences, P. O. Box 1129, Hefei 230031 (P. R. China); 2 University of Science & Technology of China, Hefei, 230026 (P. R. China); 3 Key Laboratory of Ion Beam Bioengineering, Chinese Academy of Sciences, P. O. Box 1138, Hefei 230031 (P. R. China)

## Abstract

Nanocontainers have great potentials in targeted drug delivery and nanospace-confined reactions. However, the previous synthetic approaches exhibited limited control over the morphology, size and materials of the nanocontainers, which are crucial in practical applications. Here, we present a synthetic approach to multi-segment linear-shaped nanopores with pre-designed morphologies inside anodic aluminium oxide (AAO), by tailoring the anodizing duration after a rational increase of the applied anodizing voltage and the number of voltage increase during Al foil anodization. Then, we achieve nanocontainers with designed morphologies, such as nanofunnels, nanobottles, nano-separating-funnels and nanodroppers, with tunable sizes and diverse materials of carbon, silicon, germanium, hafnium oxide, silica and nickel/carbon magnetic composite, by depositing a thin layer of materials on the inner walls of the pre-designed AAO nanopores. The strategy has far-reaching implications in the designing and large-scale fabrication of nanocontainers, opening up new opportunities in nanotechnology applications.

Nanocontainers (containers with their inner cavities at least one dimension in nanoscaled size), such as natural halloysite nanotubes^1^, have great potentials in holding nanogram quantities of materials^2^, targeted drug delivery^3^, confined (bio-) chemical reactions^4^ and nanometrology^2^. Although various nanocontainers, such as polymersomes^5^ and micelles^3^, carbon nanohorns^6^, protein capsids^7^, gold nanospheres^8^, and mesoporous silica matrices^9^, have been achieved, the present synthetic approaches demonstrate very limited control over the morphology, size and materials of the nanocontainers, which are very important in the nanocontainer applications^4^,^9^,^10^. Fabrication inside rationally designed porous anodic aluminium oxide (AAO) templates is ideal to produce large quantities of nanocontainers with regular uniform morphologies and tunable sizes, but this feat has been accomplished only for nanoscaled test tubes^11^ and cups^2^. Herein, we rationally increase the applied anodizing voltage during the Al foil anodization and tune the anodizing duration after the voltage increase as well as the number of the voltage increase to tailor the shape of the nanopores inside the porous AAO template (as shown schematically in Fig. 1a), and then deposit a uniform thin layer of materials on the inner pore walls of the AAO to build nanocontainers with pre-designed morphologies, sizes and materials. Using the approach, we have achieved four new types of nanocontainers, i.e., nanofunnels, nanobottles, nano-separating-funnels and nanodroppers (see Fig. 1b for each of them), with different sizes and diverse materials such as carbon, silicon, germanium, hafnium oxide, silica and nickel/carbon magnetic composite.

## Results

Nanoporous AAO template has been studied for over 60 years, and it is known that the diameter of the nanopores inside the AAO is directly proportional to the applied anodizing voltage^12^, so the pore diameter and the pore morphology can be tailored by tuning the applied anodizing voltage during the anodization of Al foil. Usually the conventional constant voltage anodization leads to hexagonally arranged and monodispersed cylindrical nanopores^12^, and decreasing the applied voltage during the anodization leads to the nanopores branching into several small-diameter pores^13^,^14^. These two types of nanopores have been widely used as templates for the synthesis of mono- and hetero-nanostructures with linear^11^ and branched topologies^14^,^15^,^16^. It was also reported that increasing the applied voltage during the Al foil anodization could transform the original mild anodization to hard anodization and lead to the original pores narrowing, thus pores with modulated diameters along their original axes could be achieved when the mild and hard anodization were consecutively performed^12^. However, relative little has been reported about the effect of anodizing voltage increase on the pore architecture during the mild anodization of Al foil. Forty years ago Wood et al.^17^ found that the original pores divided into two types of pores after the increase of anodizing voltage: one type of pores grew for a short period of time and then stopped growing forever (denoted as “dead pores”); while the other type of pores grew continuously and ultimately had a large stable diameter (denoted as “growing pores”). Recently, Ruoff's group^18^ and we^19^ found that the “growing pores” could still be regularly arranged only if the anodizing voltage was increased by a given factor of 2^18^ or 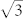
19 in the Al foil anodization.

**Figure 1:**
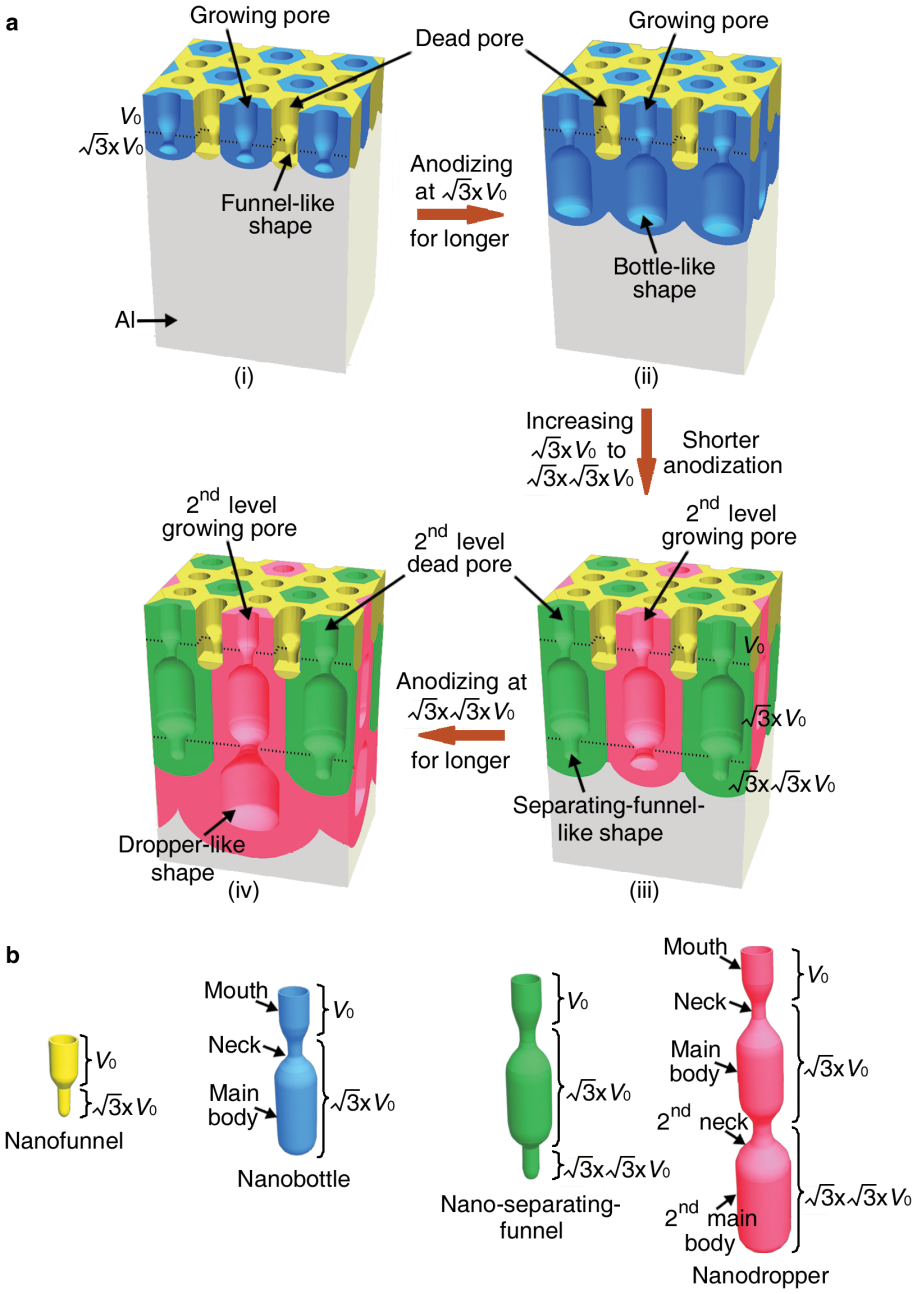
Schematics for the new four types of nanopores and nanocontainers. (a) Fabrication of AAO with funnel-like (i), bottle-like (ii), separating-funnel-like (iii) and dropper-like shaped nanopores (iv). For clarity, pores of different morphologies with their corresponding hexagonal cells^18^ are shown in different colors. 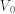 is the original anodizing voltage, and interfaces of AAO formed at different anodizing voltages are indicated by black dotted lines. (b) The four types of nanocontainers of a nanofunnel, a nanobottle, a nano-separating-funnel and a nanodropper. For each segment of the nanocontainers, the anodizing voltage for its corresponding AAO pore segment is marked on its right.

**Figure 2:**
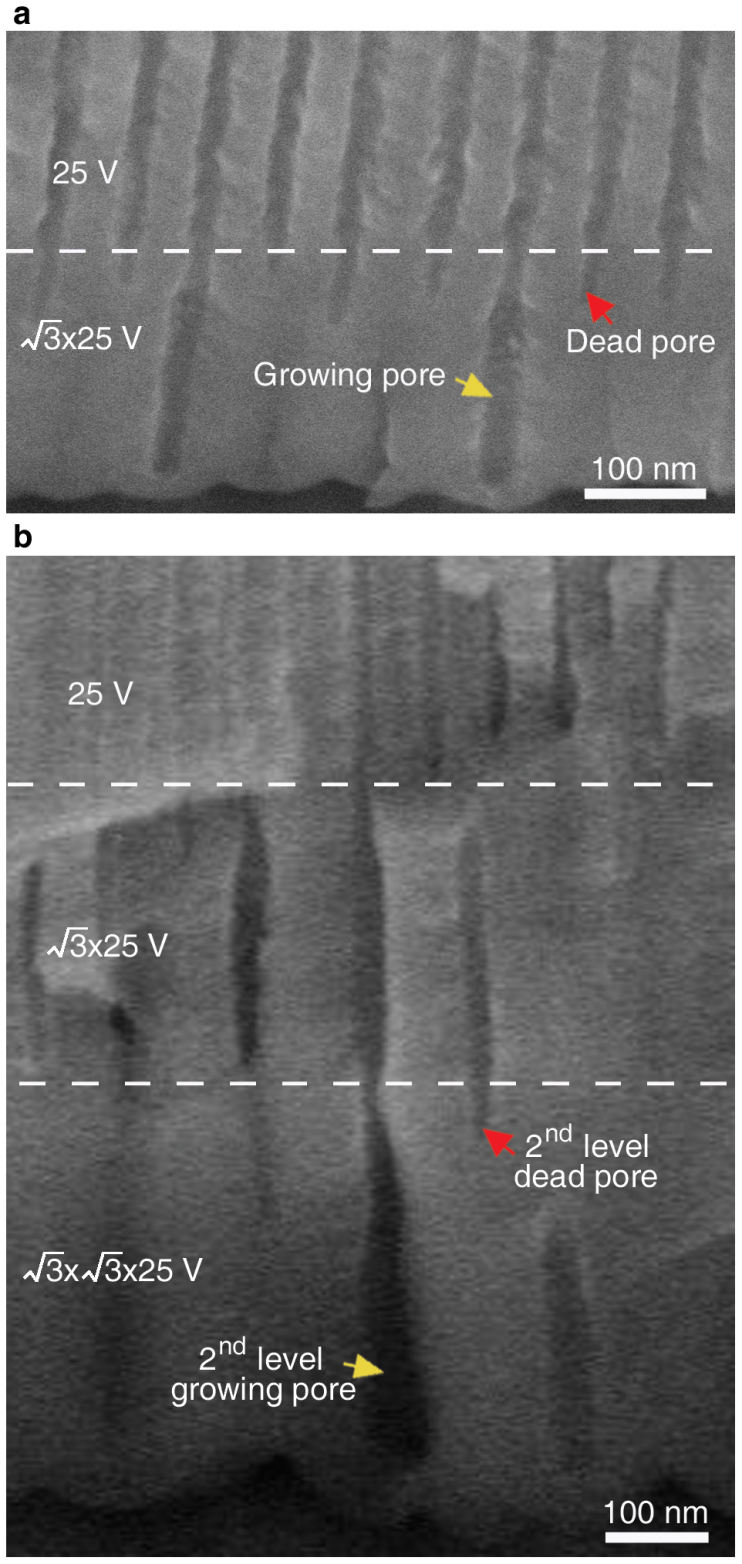
Cross-sectional SEM images of AAO templates with pre-designed multi-segment linear-shaped pores. (a) The AAO achieved by first anodizing at 25 V (above the white dotted line) and then anodizing at 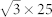
 V (below the white dotted line), showing “dead pore” and “growing pore”. (b) The AAO achieved by further continuously anodizing at 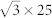
 V (between the two white dotted lines) and finally anodizing at 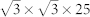
 V (below the lower white dotted line), showing the 2^nd^ level “dead pore” and “growing pore”.

On the basis of the above-mentioned new discovery, now we have developed a generic synthetic approach to four types of multi-segment linear-shaped nanopores with each type having several segments of different diameters, via rationally increasing the applied anodizing voltage by a factor of 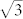 once and then twice, together with tuning the anodizing duration after the increase of the applied anodizing voltage, as shown schematically in Fig. 1a. The idea of creating these new types of pre-designed multi-segment nanopores is first proved by our scanning electron microscopy (SEM) observation on the cross-sectional morphology of the AAO nanopores achieved by increasing the applied voltage from 25 to 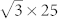
 V (Fig. 2a) and further from 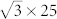 to 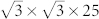
 V (Fig. 2b). It can be seen that both the “growing pores” and the 2^nd^ level “growing pores” (i.e., “growing pores” after increasing the anodizing voltage twice) have smaller diameters after the initial increase of the anodizing voltage by a factor of 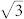. This pore narrowing can be attributed to the H^+^ activity enhancement at the original pore bottom when the applied voltage is increased^20^. Then the diameters of the “growing pores” and the 2^nd^ level “growing pores” gradually increase with the anodization going on, reach a large stable diameter, and maintain this stable diameter in further anodization. It is noted that both the “dead pores” and the 2^nd^ level “dead pores” (i.e., “dead pores” after increasing the anodizing voltage twice) have smaller diameters after the initial increase of the anodizing voltage, and then keep their small diameters until their growth termination. Therefore, suddenly increasing the applied anodizing voltage from the original anodizing voltage 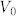 to 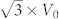 in Al foil anodization and maintaining the increased voltage of 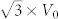 for an appropriate short period of time, the “dead pores” (two thirds of the original pores^19^) with a funnel-like shape (upper-segment-large and lower-segment-small) and the “growing pores” (one third of the original pores^19^) with their lower-segment diameter gradually increasing could be achieved, as shown schematically in Fig. 1a i. By elongating the anodization at the increased voltage of 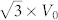, the “dead pores” are still dead with a funnel-like shape, while the “growing pores” further grow downwards wide and then maintain a large stable diameter, resulting in bottle-like shaped nanopores (Fig. 1a ii). Similarly, by further increasing the anodizing voltage from 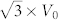 to 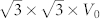 for an appropriate short period of anodization (Fig. 1a iii) and a long period of anodization (Fig. 1a iv), two thirds of the above-mentioned “growing pores” (or two ninths of the original pores) will become newly-formed 2^nd^ level “dead pores” with a separating-funnel-like shape (Fig. 1a iii) and one third of the above-mentioned “growing pores” (or one ninth of the original pores) will become newly-formed 2^nd^ level “growing pores” with a dropper-like shape (Fig. 1a iv), respectively. Using these four types of multi-segment nanopores with pre-designed shapes inside the AAO as templates, we deposit on the inner pore walls a uniform thin layer of various materials that exactly duplicate the pore morphology and size^21^,^22^,^23^,^24^. After selectively etching the AAO template, large quantities of nanocontainers with four types of morphologies (Fig. 1b shows each type schematically), i.e., nanofunnels, nanobottles, nano-separating-funnels and nanodroppers, could be achieved. Theoretically, any materials that were previously fabricated in the nanopores of AAO template as nanotubes could be built inside our new pre-designed multi-segment and linear-shaped nanopores, to achieve the new four types of nanocontainers.

**Figure 3:**
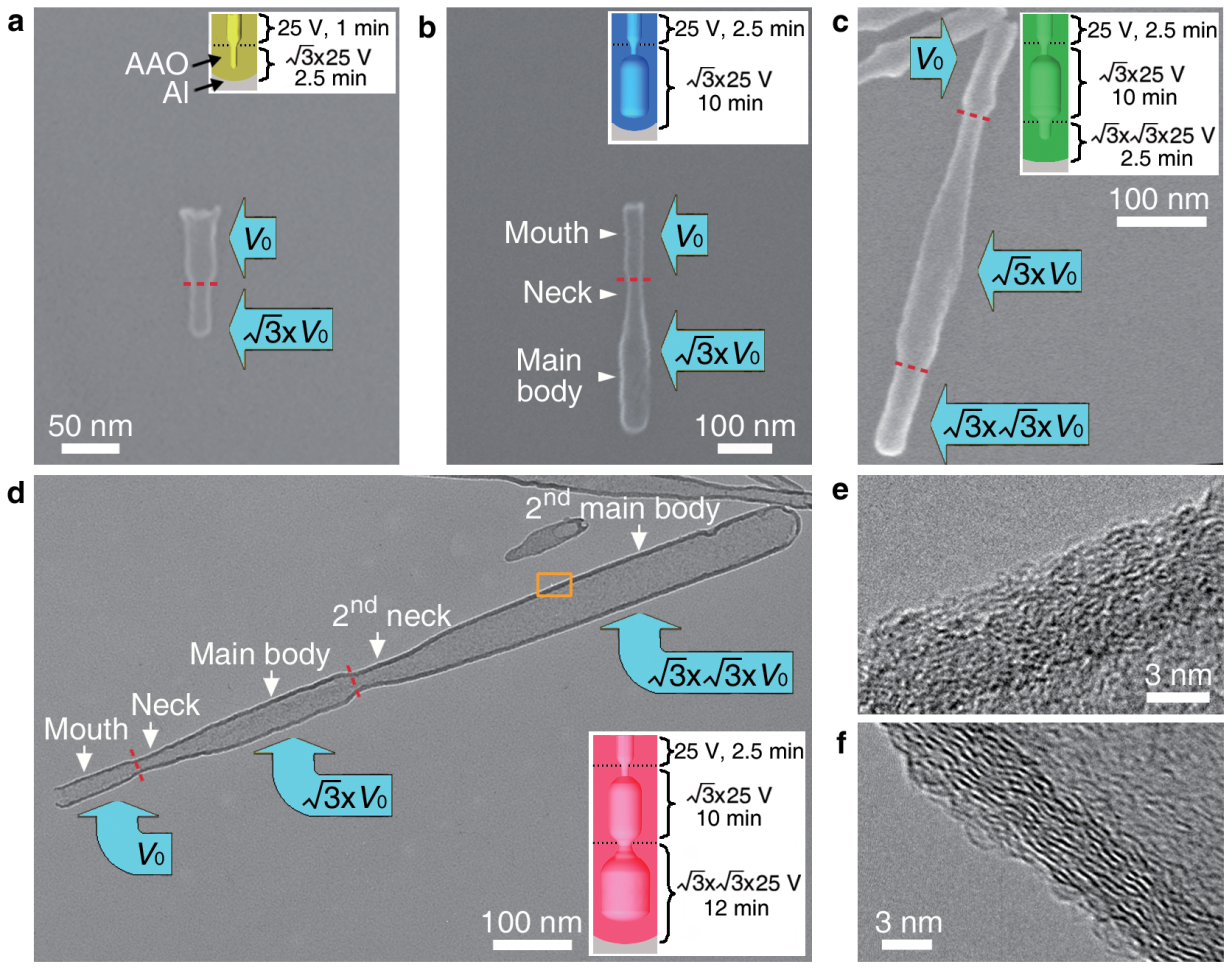
Structural characterization of the four types of carbon nanocontainers. (a–c) SEM images of a carbon nanofunnel, a carbon nanobottle and a carbon nano-separating-funnel, respectively. (d) TEM image of a carbon nanodropper. (e) HRTEM image of the carbon nanodropper wall taken from the rectangular area marked in (d). (f) HRTEM image of the carbon nanodropper wall, where CVD was conducted at 800°C. The insets in (a–d) show schematics of AAO pores used for the synthesis of the carbon nanocontainers, with anodizing conditions marked on the right of the pores.

Firstly, we achieved the four types of nanopores with pre-designed morphologies inside the AAO templates by applying an anodizing voltage of 25 V as the original voltage. Then we deposited a thin carbon layer on the inner pore walls of these AAO templates to achieve nanocontainers made of carbon, by pyrolysis of acetylene in an AAO self-catalyzed chemical vapor deposition (CVD) process without using any catalysts^21^. After selectively wet-chemically etching the AAO templates, large quantities of carbon nanocontainers have been achieved (Supplementary Fig. S1 and Supplementary Text S1). Detailed structural characterization of the four types of nanocontainers is shown in Fig. 3. Figure 3a is a SEM image of a typical carbon nanofunnel, revealing two segments of different diameters, with each segment having a relatively uniform diameter. The upper wide segment is ~65 nm long with an open mouth upwards and a diameter of ~30 nm, being well in agreement with those of the AAO nanopores achieved with the original anodizing voltage of 25 V for 1 min. The lower narrow segment is ~50 nm long with a closed end at the bottom and a diameter of ~20 nm, being the duplication of those of the dead pores achieved with an increased anodizing voltage of 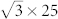
 V for an appropriate anodizing duration of 2.5 min. Figure 3b is a representative SEM image of a carbon nanobottle, revealing three segments of short “mouth” (the upper segment), the “neck” (the middle segment) and the “main body” (the lower segment), respectively. The short “mouth” segment is ~130 nm long with an open mouth upwards and a uniform diameter of ~30 nm, being the duplication of the AAO nanopore achieved with the original anodizing voltage of 25 V for 2.5 min. The middle “neck” segment is ~130 nm long with its diameter gradually increasing from the end of the “mouth” to the beginning of the “main body”, being the duplication of the growing pore segment achieved in the initial anodization stage at the increased voltage of 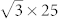
 V. While the “main body” segment is ~150 nm long with a closed end at the bottom and a uniform diameter of ~45 nm, corresponding well with the nanopore segment achieved in the stable anodization at 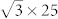
 V. In comparison with the nanobottle shown in Fig. 3b, the carbon nano-separating-funnel shown in Fig. 3c has one more segment (i.e., the bottom end segment) of ~100 nm length and with a smaller diameter of ~35 nm, and this additional segment is the duplication of the 2^nd^ level “dead pore” end segment resulting from the anodization at 75 V (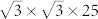
 V) for 2.5 min after the 2^nd^ time increase of the anodizing voltage. From Fig. 3b and the transmission electron microscopy (TEM) image in Fig. 3d, it can be seen that the carbon nanodropper (Fig. 3d) has two more segments than the carbon nanobottle, i.e., the 2^nd^ “neck” and the 2^nd^ “main body”. These two more segments together are ~600 nm long, with the 2^nd^ “main body” having a uniform diameter of ~60 nm and a closed end. The geometrical morphologies of these two more segments are the duplications of the 2^nd^ level “growing pore” segment achieved by anodizing at 75 V (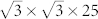
 V) for 12 min after the 2^nd^ time anodizing voltage increase. Figure 3d also reveals that the carbon nanocontainers have a uniform wall thickness of ~5 nm, and the inner cavities of the dropper-like shape can be seen clearly. High-resolution TEM (HRTEM) observation indicates that the wall of the carbon nanocontainers has low crystallinity after the CVD growth (Fig. 3e), being consistent with the previous report^18^. However, the carbon wall crystallinity can be much improved by a higher temperature (800°C) CVD growth^25^, which is confirmed by the discontinued and roughly parallel graphene layers shown in Fig. 3f.

It should be noted that not only the morphology but also the size in each segment of the nanocontainers can be tailored by simply tuning the anodizing voltages in the Al foil anodization. As the pore diameter is directly proportional to the applied anodizing voltage^12^, therefore four types of nanocontainers with each segment having a larger diameter could be obtained by using similar AAO template achieved with a larger initial anodizing voltage of 40 V (Supplementary Fig. S2a–d and Supplementary Text S2). On the other hand, the diameters in all segments of the nanocontainers can be increased to some extent as the AAO pores can be isotropically widened by etching in phosphoric acid solution^21^. For example, carbon nanodroppers, where diameter of each segment is larger than that of the corresponding segment of the carbon nanodropper in Supplementary Figure S2d, have been realized by using the pre-designed AAO with additional etching in 1 M phosphoric acid for 20 min as template (Supplementary Fig. S2e and Supplementary Text S3).

Then, we tried to extend our pre-designed AAO pore confined CVD growth to the four types of nanocontainers made of other materials rather than carbon. As a crystalline Si thin uniform layer can be deposited on the inner pore walls of AAO by pyrolysis of silane in a CVD process^22^, we performed the similar CVD process using our new AAO with pre-designed nanopores as template, and achieved the four types of nanocontainers made of Si. For example, crystalline Si nanobottles have been achieved (Supplementary Fig. S3). Afterwards, we tried to build Ge nanocontainers by depositing a thin uniform layer of crystalline Ge on the inner pore walls of AAO with pre-designed nanopores, by pyrolysis of germane in a Ni nanoparticles catalyzed CVD process. As expected, crystalline Ge nano-separating-funnels have been obtained (Supplementary Fig. S4).

Next, we tried to construct nanocontainers made of other materials by using other techniques rather than CVD growth. As atomic layer deposition (ALD) can produce conformal thin films with precise thickness control at the atom scale^23^, we applied ALD technique to deposit a very thin layer of HfO_2_ on the inner pore walls of our AAO with pre-designed nanopores to achieve nanocontainers made of HfO_2_. As expected, HfO_2_ nanobottles have been achieved (Supplementary Fig. S5). Then we tried to build SiO_2_ nanocontainers due to their biological stability and easy modification by surface chemistry^10^. As SiO_2_ thin layer could be deposited on the inner pore wall of AAO by repeated dipping^24^, we performed the similar process using our new AAO with pre-designed nanopores. As a result, SiO_2_ nanocontainers have been achieved (e.g., SiO_2_ nanodroppers shown in Supplementary Fig. S6). In addition to all those methods mentioned above, other approaches such as wetting^26^, layer-by-layer deposition^27^, and electroless plating^28^, may also be exploited for the building of our new four types of nanocontainers made of polymer^26^, protein^27^, and metal^28^, respectively.

**Figure 4:**
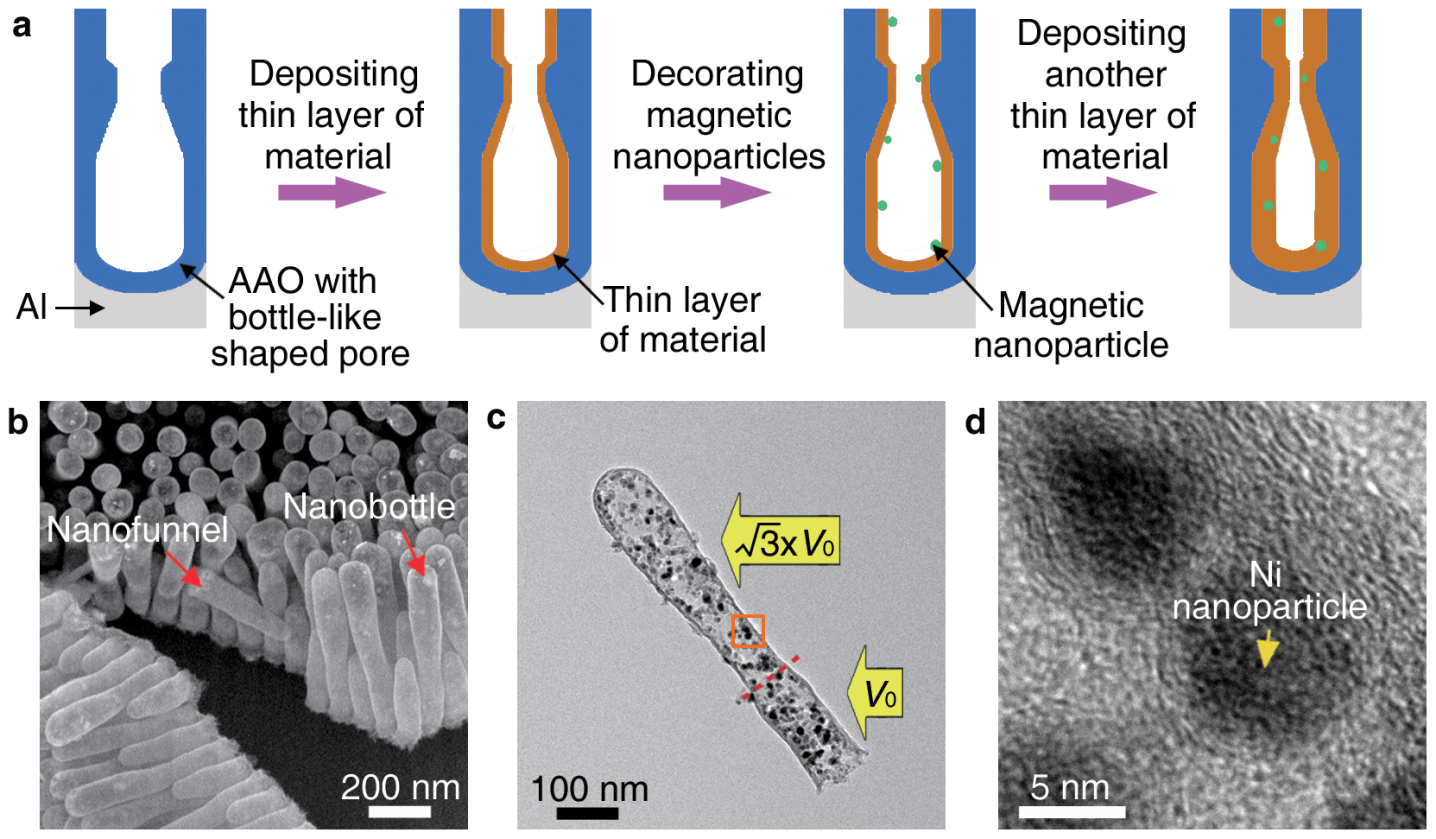
Nanocontainers with their walls embedded with magnetic nanoparticles. (a) Schematic for embedding magnetic nanoparticles within the nanocontainer wall. (b) SEM image of carbon nanofunnels and nanobottles, with walls embedded with Ni nanoparticles. (c) TEM image of a Ni-nanoparticle-embedded carbon nanobottle. (d) HRTEM image of the Ni-nanoparticle-embedded carbon nanobottle wall taken from the square area marked in (c).

Finally, for the easy guidance or manipulation of the nanocontainers in practical applications and the collection afterwards, we tried to embed magnetic nanoparticles within the nanocontainer wall to achieve nanocontainers made of magnetic composites, where unwanted (bio-) chemical interactions between magnetic nanoparticles and substances filled into nanocontainer inner cavities can be avoided^29^. This could be accomplished by firstly depositing a very thin uniform layer of materials on the inner walls of the pre-designed AAO nanopores, then decorating very small magnetic nanoparticles onto the pre-deposited thin layer of materials, and finally depositing another thin uniform layer of the same or different materials to cover the magnetic nanoparticles, as shown in Fig. 4a schematically. For example, for carbon nanocontainers embedded with Ni nanoparticles in their walls (Fig. 4b), we deposited a thin layer of carbon on the inner walls of the pre-designed AAO nanopores using the above-mentioned CVD growth, then decorated Ni nanoparticles onto the pre-grown carbon layer using nickel nitrate decomposition (which led to achievement of nickel oxide) and the following nickel oxide reduction, and finally deposited another thin uniform carbon layer to sheath the small Ni nanoparticles using a low temperature CVD growth (to prevent the Ni-catalyzed growth of carbon nanotubes^30^). Fig. 4c,d and Supplementary Figure S7a show that Ni nanoparticles are indeed embedded within the walls of the carbon nanocontainers. These Ni-nanoparticle-embedded carbon nanocontainers would be manipulated in practical applications such as drug delivery by using a magnetic field, and even might be collected afterwards (Supplementary Fig. S7b).

## Discussion

We have demonstrated a generic synthetic approach to large quantities of nanocontainers of nanofunnels, nanobottles, nano-separating-funnels and nanodroppers, with tunable sizes and diverse materials, by using porous AAO templates with pre-designed pores, which are achieved via tailoring the anodizing duration after a rational increase of the applied anodizing voltage and the number of voltage increase during the Al foil anodization. These are, to our knowledge, the first reported results in controlled large-scale building of nanocontainers made of different materials with delicately tailored morphologies and sizes, and the four types of unique nanocontainers may open up new opportunities for both fundamental research and practical applications. For example, the multi-segment nanocontainers with different segments having different diameters are ideal nanoreactors to study the effect of different spatial confinements on (bio-) chemical reactions^4^. In addition, nanobottles, nano-separating-funnels and nanodroppers, which have smaller diameter in their neck segments, are particularly promising in applications such as sustained-release drug-delivery systems, as the narrowed openings of nanocontainers favor the constrained diffusion of drugs within the nanocontainer inner cavities^9^.

## Methods

### Fabrication of AAO with nanopores having four types of pre-designed shapes

AAO templates with nanopores having four types of pre-designed shapes were fabricated by following the two-step anodization process^31^. For AAO with funnel-like or bottle-like shaped pores, the anodizing voltage was increased by a factor of 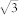 once, and for that with separating-funnel-like or dropper-like shaped pores, the anodizing voltage was increased by a factor of 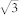 twice. For anodization at 25, 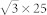 and 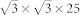
 V, 0.3 M sulfuric acid, 0.3 M oxalic acid and 0.04 M oxalic acid were used, respectively, and for that at 40, 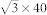 and 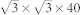
 V, 0.3, 0.04 and 0.01 M oxalic acid were used, respectively. In addition, the acid etching was performed in 1 M phosphoric acid at 30°C.

### Growth of carbon, Si and Ge nanocontainers

The carbon nanocontainers were grown inside AAO pores with four types of pre-designed shapes by pyrolysis of C_2_H_2_ at 650°C for 2 h, with C_2_H_2_ and Ar of 3 and 125 s.c.c.m., respectively^21^. For growth of carbon nanocontainers with improved wall crystallinity, the pyrolysis of C_2_H_2_ was conducted at 800°C for 7 min, with C_2_H_2_ and Ar of 3 and 225 s.c.c.m., respectively^25^.

The growth of Si nanocontainers was achieved by pyrolysis of SiH_4_ at 500°C for 1 h, with SiH_4_, N_2_ and H_2_ of 3.5, 100 and 15 s.c.c.m., respectively, followed by in situ annealing at 750°C for 2 h, with N_2_ and H_2_ of 100 and 15 s.c.c.m., respectively^22^.

For growth of Ge nanocontainers, we first decorated nickel nitrate onto inner pore walls of AAO with pre-designed nanopores by immersing AAO in 0.12 M nickel nitrate aqueous solution and then drying the AAO in air. Then, we annealed the AAO at 330°C for 2 h, with Ar and H_2_ of 60 and 15 s.c.c.m., respectively. During this process, the decomposition of nickel nitrate led to the formation of nickel oxide nanoparticles, which were then reduced to Ni nanoparticles by H_2_. After the annealing process, we grew Ge nanocontainers by pyrolysis of GeH_4_ at 330°C, with Ni nanoparticles as catalysts and GeH_4_, Ar and H_2_ of 30, 60 and 15 s.c.c.m., respectively. Finally, we annealed the Ge nanocontainers at 400°C, with Ar and H_2_ of 60 and 15 s.c.c.m., respectively.

### Growth of HfO_2_ nanocontainers

The growth of HfO_2_ nanocontainers was achieved by using ALD approach^23^, where deposition of HfO_2_ was conducted at 250°C, with Hf(NMe_2_)_4_ (pulse length: 0.3 s), deionized water (pulse length: 0.3 s), and N_2_ (20 s.c.c.m.) as organometallic precursor, oxidant, and carrying gas, respectively. Then, we annealed the HfO_2_ nanocontainers at 650°C for 1 h in air.

### Growth of SiO_2_ nanocontainers

We grew SiO_2_ nanocontainers by using the approach of repeated dipping^24^, where deposition of SiO_2_ onto inner pore walls of AAO with pre-designed pores was achieved by hydrolysis of SiCl_4_. Firstly, AAO with pre-designed nanopores was immersed in a mixture of SiCl_4_ (12 ml) and CCl_4_ (20 ml) for 2 min, followed by quickly washing the AAO with CCl_4_. Then, the AAO was sequentially soaked in CCl_4_, mixture of CCl_4_ and methanol (1:1), and ethanol for 27, 2 and 4 min, respectively, followed by annealing in Ar at 110°C for 3 h. After the annealing process, the AAO was immersed sequentially in deionized water and methanol for 5 and 2 min, respectively, followed by the second annealing process.

### Growth of Ni-nanoparticle-embedded carbon nanocontainers

Firstly, thin uniform layer of carbon was deposited onto inner pore walls of AAO with pre-designed nanopores via the 1^st^ CVD process^21^, where pyrolysis of C_2_H_2_ was conducted at 650°C for 2 h, with C_2_H_2_ and Ar of 3 and 125 s.c.c.m., respectively. Then, the AAO was subjected to plasma cleaning to make the carbon layer hydrophilic^21^, followed by immersing AAO in 1 M nickel nitrate aqueous solution, drying the AAO and annealing the AAO at 450°C with Ar and H_2_ of 60 and 15 s.c.c.m., respectively. During the annealing process, the decomposition of nickel nitrate led to the formation of nickel oxide nanoparticles, which were then reduced to Ni nanoparticles by H_2_. Finally, the 2^nd^ CVD process, where pyrolysis of C_2_H_2_ was conducted at 450°C for 2 h, with C_2_H_2_ and Ar of 20 and 50 s.c.c.m., respectively, was carried out.

### Characterization of nanocontainers

AAO templates with pores embedded with carbon or HfO_2_ nanocontainers or Ni-nanoparticle-embedded carbon nanocontainers were dissolved by 3 M NaOH at 60°C for 3 h (for carbon nanocontainers grown at 800°C, the AAO was dissolved by 10 M NaOH at 85°C for 24 h), and those with pores embedded with Si or SiO_2_ nanocontainers were dissolved by concentrated HCl solution at 85°C for 10 h. In addition, AAO templates with pores embedded with Ge nanocontainers were dissolved by 1 M NaOH at room temperature for 8 h. After dissolving the AAO, the liberated nanocontainers were rinsed by deionized water for several times, followed by dispersing them in ethanol. Finally, the nanocontainers were characterized by using SEM (Sirion 200, FEI, at 5 KV) and TEM (JEM 2010, at 200 KV) with selected area electron diffraction (SAED) and energy disperse X-ray spectrum (EDS).

## Author Contributions

X. Z. and G. M. designed the experiments. X. Z. carried out most of the experiments (fabrication of AAO templates, growth of carbon and HfO_2_ nanocontainers). F. H. drew the schematics and fabricated the SiO_2_ nanocontainers. X. L. conducted the growth of Ge nanocontainers. B. C. worked on the growth of Si nanocontainers. X. Z. (Xiaoguang Zhu) and Z. C. carried out the TEM experiments. M. K. carried out the SEM experiments. All authors discussed the results and analysed the data. X. Z., G. M., Q. H. and Q. X. co-wrote the manuscript. G. M. supervised the work.

## Supplementary Material

Supplementary Information Supplementary information for Metal hierarchical patterning by direct nanoimprint lithography
