# Supplementary material for: Nanocontainers made of Various Materials with Tunable Shape and Size
Source: Sci Rep. 2013 Jul 19;3:2238. doi: 10.1038/srep02238 (PMC3715783; doi:10.1038/srep02238)
Supplement: Supplementary Information — for Metal hierarchical patterning by direct nanoimprint lithography [file srep02238-s1.pdf]

Supplementary information for

Metal hierarchical patterning by direct nanoimprint lithography

Boya Radha[1,2], Su Hui Lim[2,3], Mohammad S. M. Saifullah[2*] and Giridhar U. Kulkarni[1*]

[1] *Chemistry and Physics of Materials Unit and DST Unit on Nanoscience, Jawaharlal Nehru Centre for Advanced Scientific Research, Jakkur P.O., Bangalore 560 064, India*

[2] *Institute of Materials Research and Engineering, A*STAR (Agency for Science, Technology and Research), 3 Research Link, Singapore 117602, Republic of Singapore*

[3] *Department of Electrical and Computer Engineering, National University of Singapore, 4 Engineering Drive 3, Singapore 117576, Republic of Singapore*

S1. TGA and DSC analysis 2
Table S1. Direct nanoimprinting conditions for various patterns of Pd 4
S2. AFM images of 250 nm lines (a) as-imprinted and (b) after heat treatment 5
S3. Optical image demonstrating large area uniform line patterns of Pd 6
S4. SEM and AFM images showing large area uniform dimple patterns of Pd 6
S5. SEM images of versatile nano/micro structures imprinted by MDNIL 7
S6. SEM images of the 100 nm Pd lines (a) as-imprinted and (b) after heat treatment 7
S7. SEM images of the nanoimprinted 35 nm lines 8
S8. Pd nanotubes 8
S9. As-imprinted Pd hexagonal arrays 9
S10. Optical image of failed hierarchical patterning of Pd 9
S11. Pd hierarchical structures 10
S12. Transfer stacking 11
S13. AFM z-image of the stacked Pd thiolate lines 12
S14. Failed transfer stacking 12
S15. Biomimicking 13
S16. Rice leaf contact angles 14
S17. Calculation of enhancement factor 14

* Correspondence and requests for materials should be addressed to G.U.K. (kulkarni@jncasr.ac.in) and M.S.M.S. (saifullahm@imre.a-star.edu.sg)

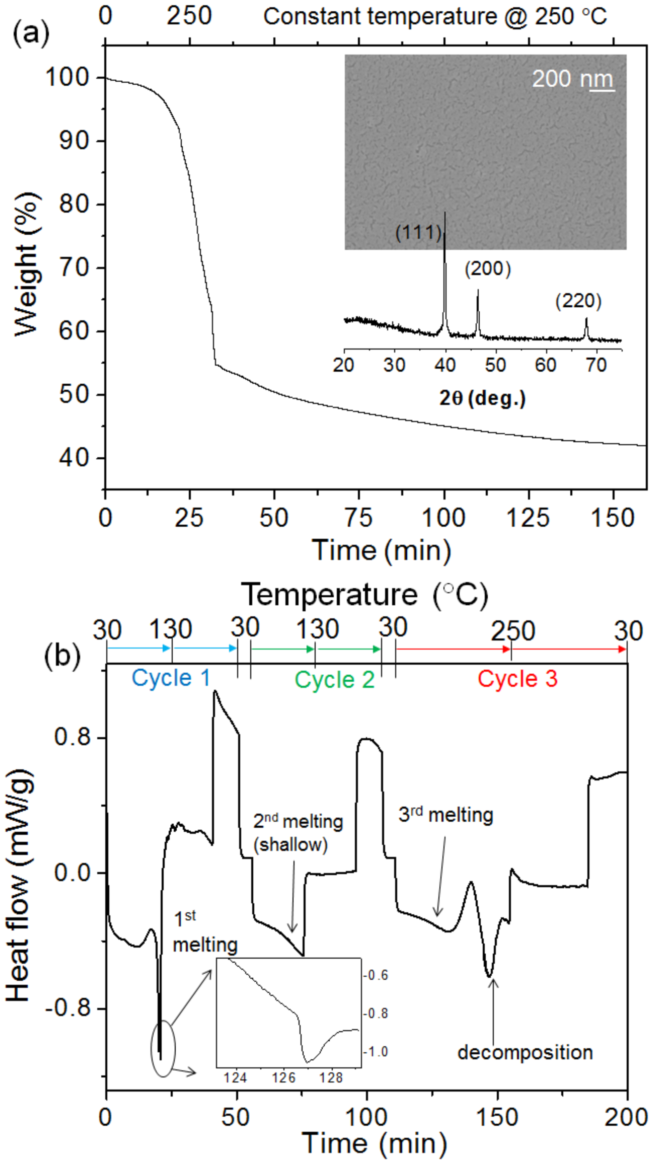

Figure S1. (a) Isothermal TGA of Pd benzylthiolate. Temperature was raised till 250 °C and held there for 60 min. SEM image and XRD of the thermolysed Pd film are shown in inset. (b) DSC of the same repeated for various melting cycles (details in the text). The first melting curve is shown enlarged in the inset.

Analogous to Tg in polymeric systems, the chosen precursor, Pd thiolate possessing crystalline lamellar structures also undergo melting [1]. In the case of linear alkanethiolates of Pd, the melting of the thiolates is well studied [1] and was observed at around 85 °C for Pd dodecylthiolate. Pd benzyl thiolate was chosen for this study, in order to increase the metal content in the final imprinted structure. Figure S1 shows the TGA of the Pd benzylthiolate where

it was heated to 250 °C, and held there for 2 hrs. There is a definite loss of material and the final residue corresponds to 43%, which roughly matches with the expected Pd metal (i.e., 45 %). Differential scanning calorimetry (DSC) measurement was performed to find out the melting temperature of the imprinting precursor. After heating to 130 °C, the compound was then cooled back to 30 °C and after a short while, the second cycle of heating was performed, which led to a DSC trace much shallower with no evident peak. In the third cycle, the peak due to melting further diminished and when the temperature was raised to 250 °C, a distinct endothermic peak was observed due to decomposition.

[1] P.J. Thomas, A. Lavanya, V. Sabareesh, G.U. Kulkarni, Self-assembling bilayers of palladiumthiolates in organic media, Proceedings of the Indian Academy of Sciences-Chemical Sciences, 113 (2001) 611-619.

[2] M.D. Malinsky, K.L. Kelly, G.C. Schatz, R.P. Van Duyne, Chain Length Dependence and Sensing Capabilities of the Localized Surface Plasmon Resonance of Silver Nanoparticles Chemically Modified with Alkanethiol Self-Assembled Monolayers, Journal of the American Chemical Society, 123 (2001) 1471-1482.

[3] J. Li, K.S. Liang, N. Camillone Iii, T.Y.B. Leung, G. Scoles, The structure of n-octadecane thiol monolayers self-assembled on Au(001) studied by synchrotron x-ray and helium atom diffraction, The Journal of Chemical Physics, 102 (1995) 5012-5028.

Table S1. Direct nanoimprinting conditions for various patterns of Pd.

Sl. No	Mold	Conc. of Pd resist (M)	Spin speed (rpm)	Thickness (nm)	Imprinting conditions			Pd feature size (nm)	Ref. (Fig.)
					Temp (°C)	Pressure (bar)	Time (sec)		
2D structures									
1	2 µm gratings	0.28	400	501	120	50	1200	1040	S3
2	250 nm gratings	0.28	3000	323	120	50	1500	100	2a-d
3	100 nm gratings	0.1	3000	135	120	40	1800	40	2i,S6
4	35 nm gratings	0.05	6000	68	120	40	2000	35	2j,k,S7
5	Dimples with 1:2 aspect ratio	0.28	3000	323	120	50	1800	85	2e-h
6	Nanotubes	0.28	3000	323	120	50	1200	100	2m,S8
7	Hexagonal hole array	0.05	6000	68	120	50	1500	23	2n,S9
Hierarchical structures									
8	250 nm on 2 µm gratings	0.28	400	501	I, 120	50	1200	1040	3a,S11
					II, 90	30	2400	50	
8	Rice leaf	0.28	400	501	I, 120	50	1200	1700	4a,S15
					II, 92	30	2400	90	
Transfer stacking									
9	Stack 1: 2 µm	0.28	1500	501	130	30	1500	1803	3b,c S12a-c
10	Stack 2: 2 µm	0.28	2000	501	130	25	1500	1705	3d,e, S12d
11	Stack 1: 250 nm	0.28	3000	501	90	40	1500	150	3f,g
Transfer imprinting to flexible polycarbonate (PC)									
12	Pd lines transferred to PC	-	-	-	150	50	600	1700	5c,d
13	Embedded Pd lines in PC	-	-	-	150	50	600	1700	5e-h

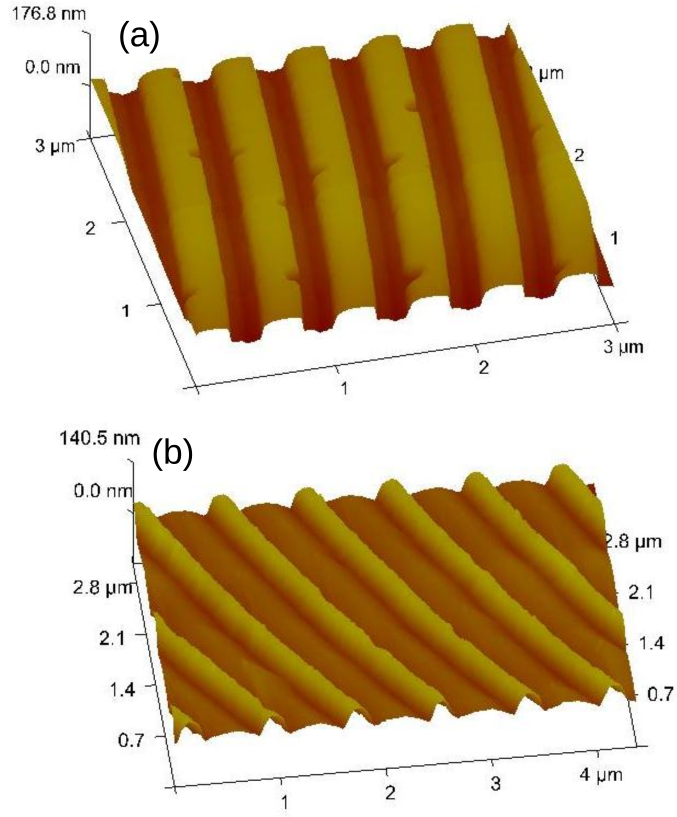

Figure S2. AFM images of 250 nm lines (a) as-imprinted and (b) after heat treatment.

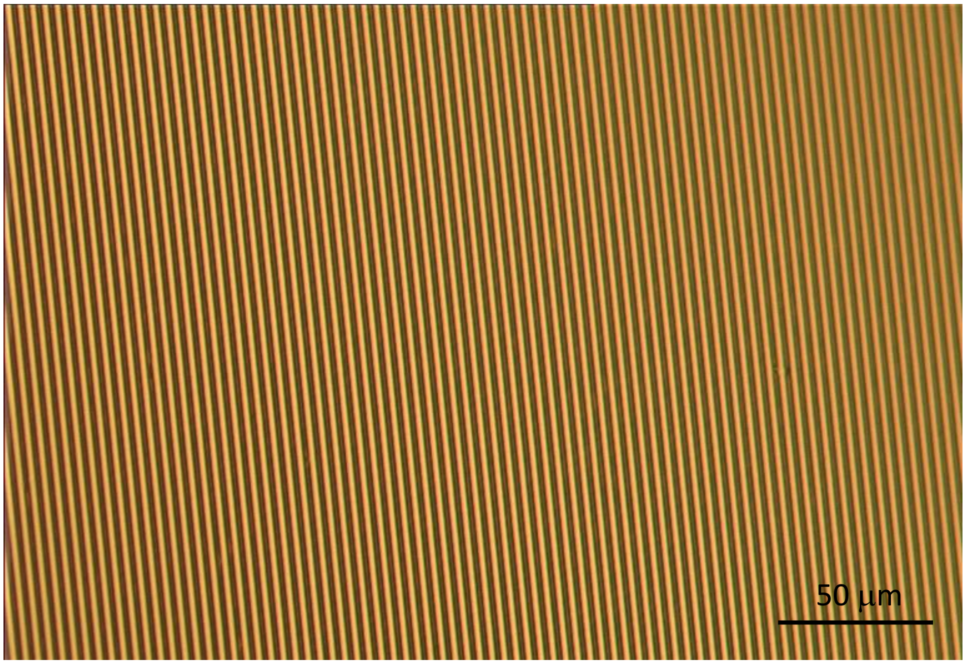

Figure S3. Optical image demonstrating large area uniform line patterns of Pd.

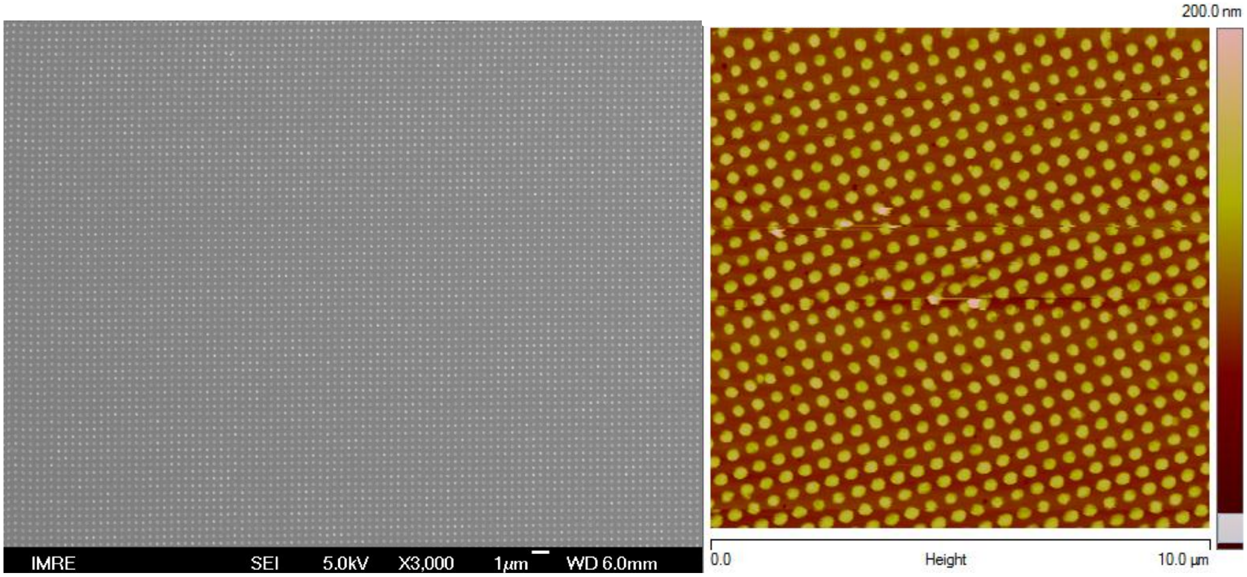

Figure S4. SEM and AFM images showing large area uniform dimple patterns of Pd.

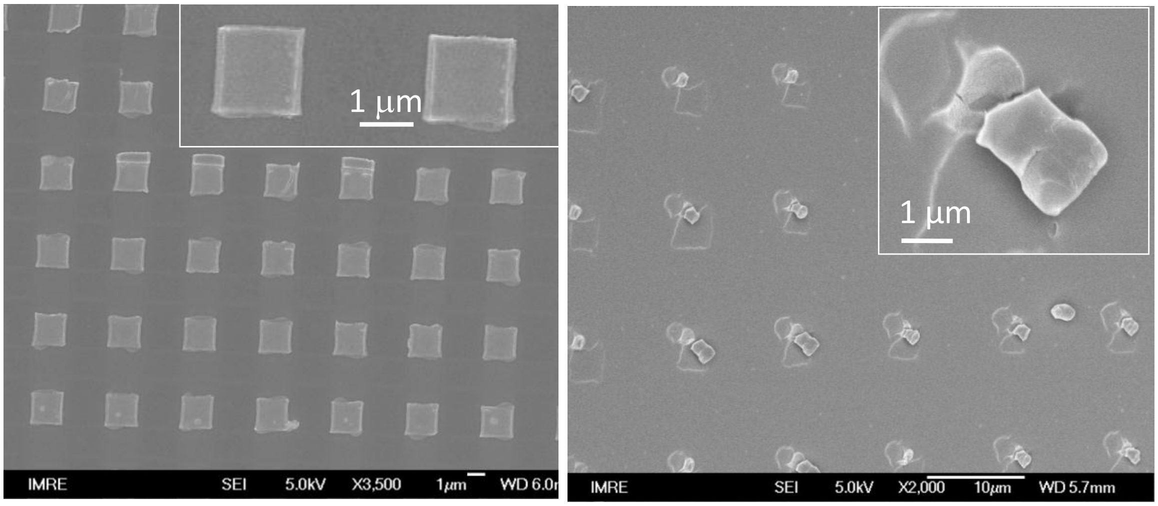

Figure S5. SEM images of versatile nano/micro structures imprinted by MDNIL

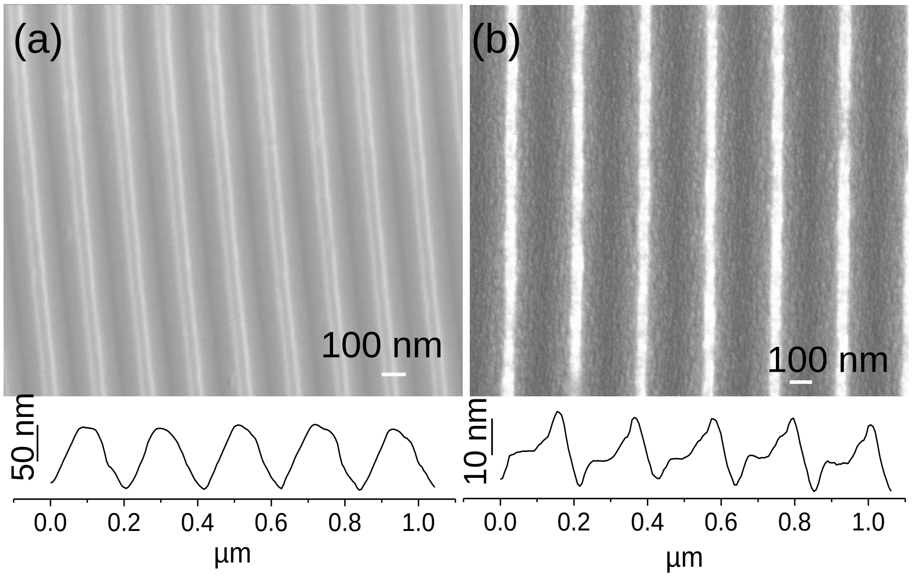

Figure S6. SEM images of the 100 nm Pd lines (a) as-imprinted and (b) after heat treatment with AFM *z*-profiles.

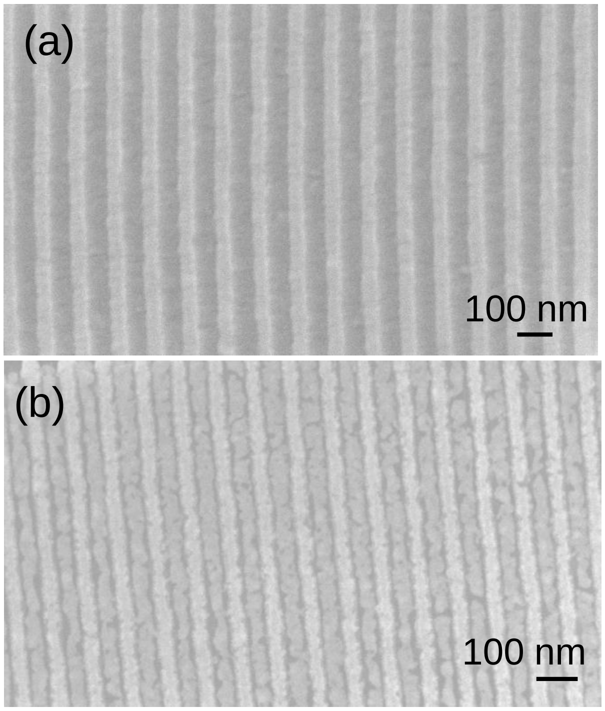

Figure S7. SEM images of the nanoimprinted 35 nm lines (a) before and (b) after heat treatment.

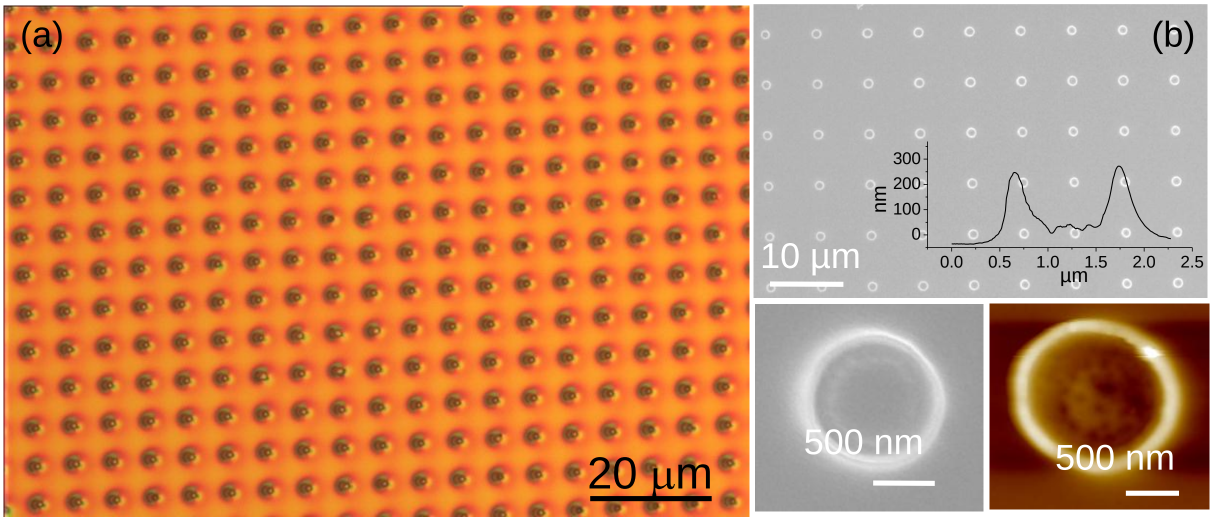

Figure S8. (a) Optical image of Pd nanotubes over large area. (b) SEM and AFM images of the same with AFM profile. The width of the wall of the as-imprinted nanotube is ~200 nm with a height of ~280 nm.

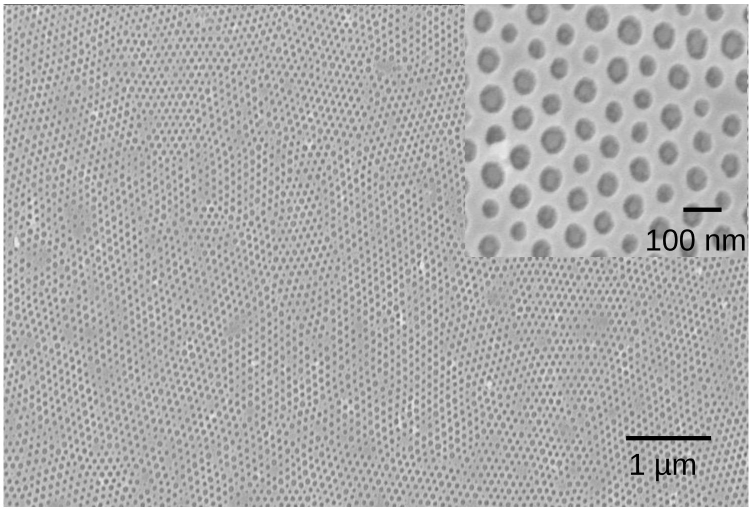

Figure S9. SEM image of the as-imprinted Pd hexagonal arrays over large area. The structure is preserved without collapsing during heat treatment.

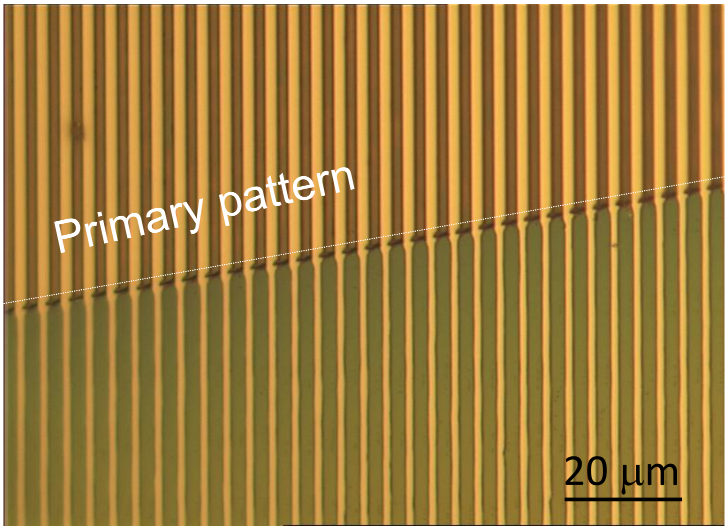

Figure S10. Optical image of failed hierarchical patterning of Pd – On the primary 2 um pattern, second imprinting with a 250 nm mold was done at an imprinting temperature of 120 °C and pressure of 50 bar. Hierarchical pattern did not form, instead the primary pattern got squashed.

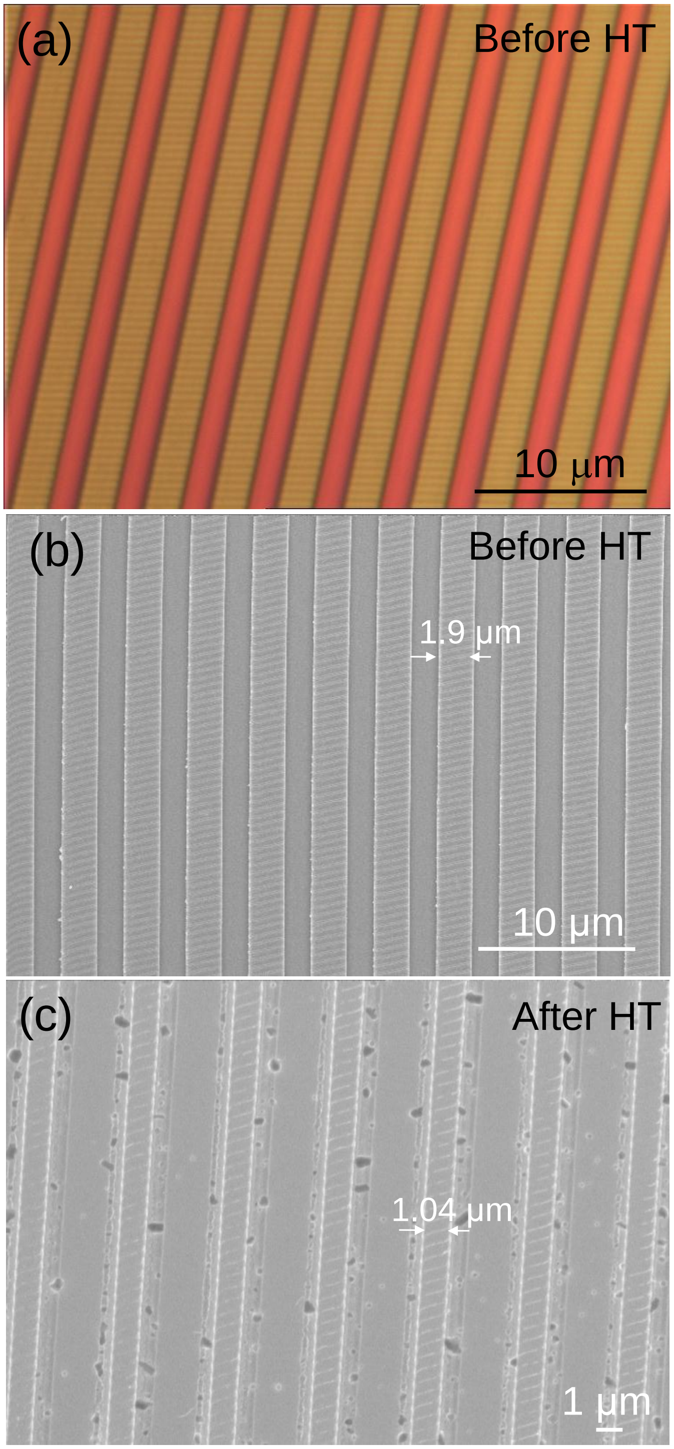

Figure S11. Optical and SEM images of Pd hierarchical structures with a primary mold of 2 μm gratings and secondary mold of 250 nm gratings. (a,b) before heat treatment and (c) after heat treatment respectively.

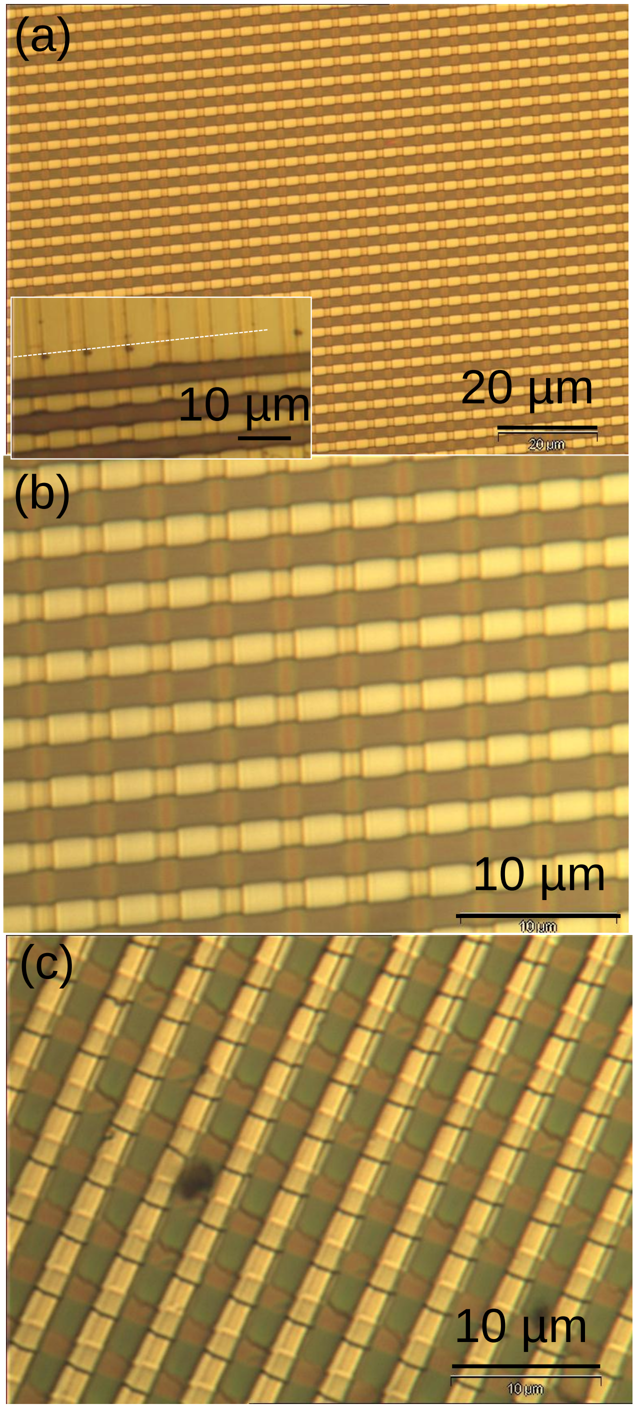

Figure S12. Transfer stacking- (a) optical image of the transfer stacked 2 µm Pd lines with the insets showing magnified views, white dotted line indicates the boundary till the secondary stacking has been performed. Optical images (b) at a higher magnification and (c) of the Pd triple stack (2 µm | 2 µm | 2 µm).

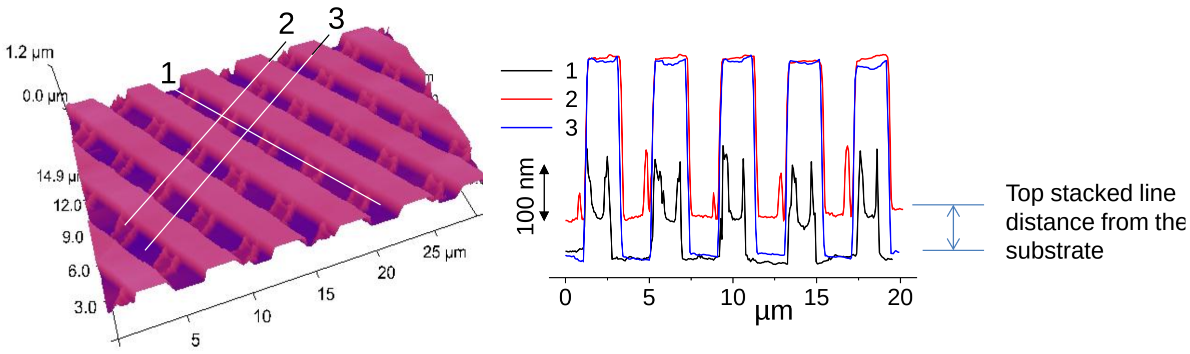

Figure S13. AFM z-image (3-dimensional) of the stacked Pd thiolate lines. Line profiles along the lines marked 1, 2 and 3 clearly reveal that the top stack is hanging on the bottom lines while not sagging towards the substrate.

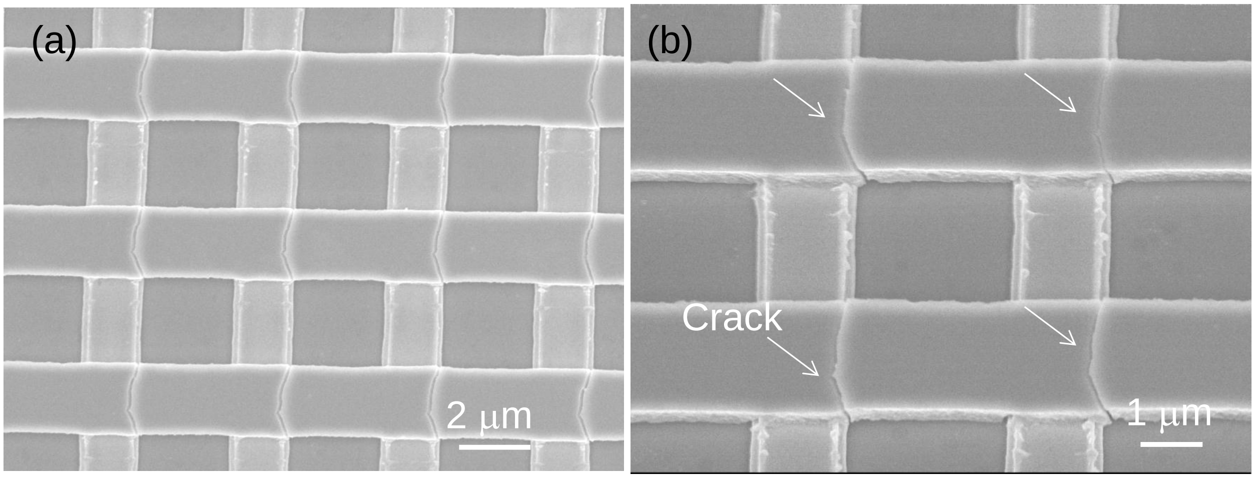

Figure S14. Failed transfer stacking - SEM images of the transfer stacked 2 µm Pd lines under a imprinting pressure of 40 bar where cracks can be seen.

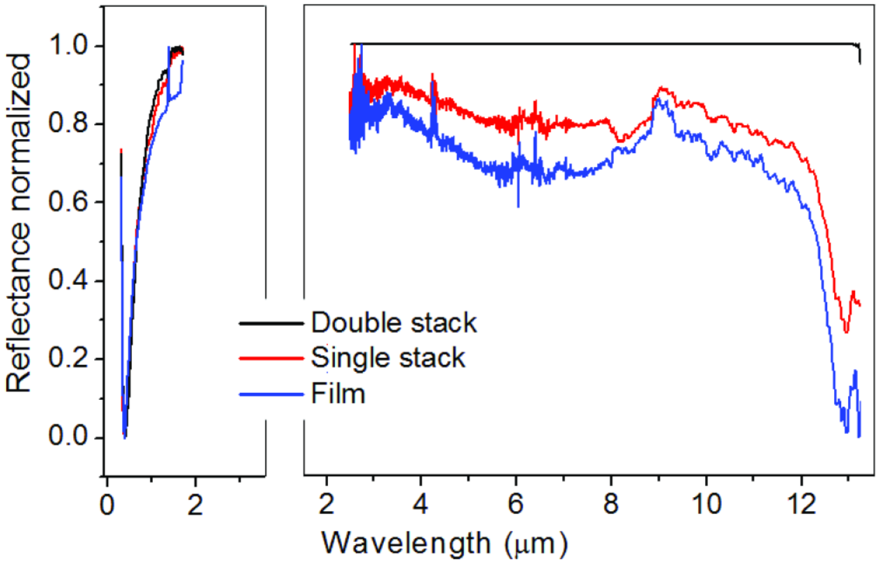

Figure S15. Normalized reflectance spectra of Pd double stack, single stack i.e., gratings and film.

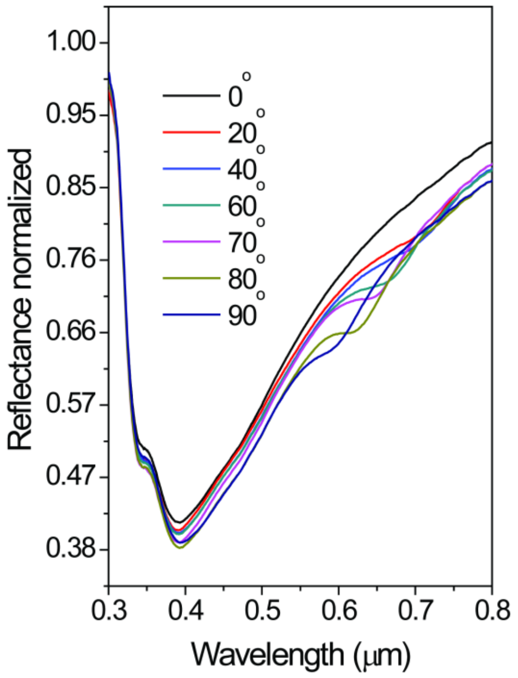

Figure S16. Angle resolved reflectance spectra of Pd stack structures in visible wavelength regime.

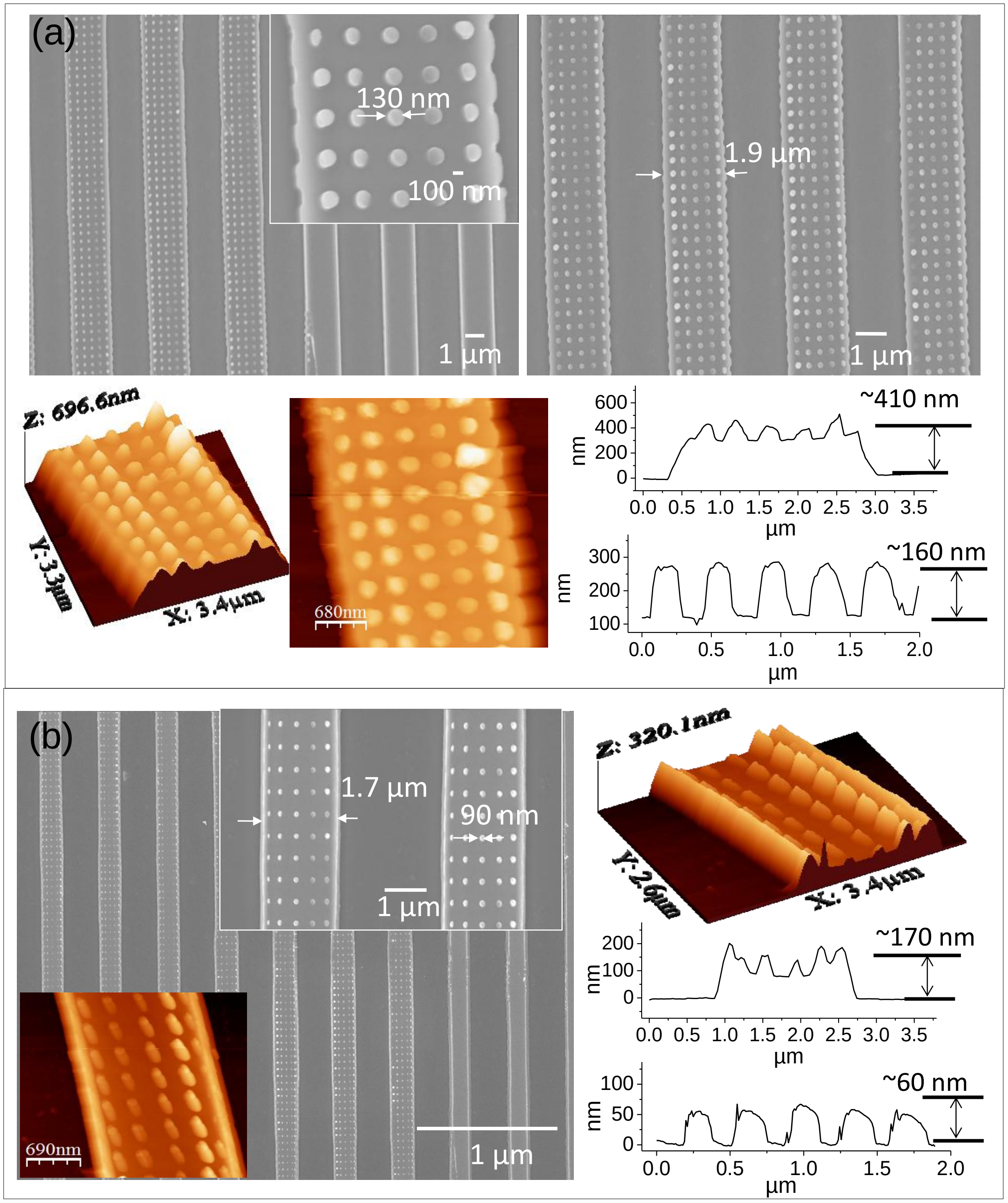

Figure S17. Biomimicking - (a) SEM images of rice leaf pattern with primary lines of 1.9 µm and secondary pillars of 130 nm, and corresponding height of 410 nm and 160 nm marked in the AFM profiles. (b) After heat treatment of rice leaf structures giving primary lines of 1.7 µm and secondary pillars of 90 nm, and corresponding height of 170 nm and 60 nm.

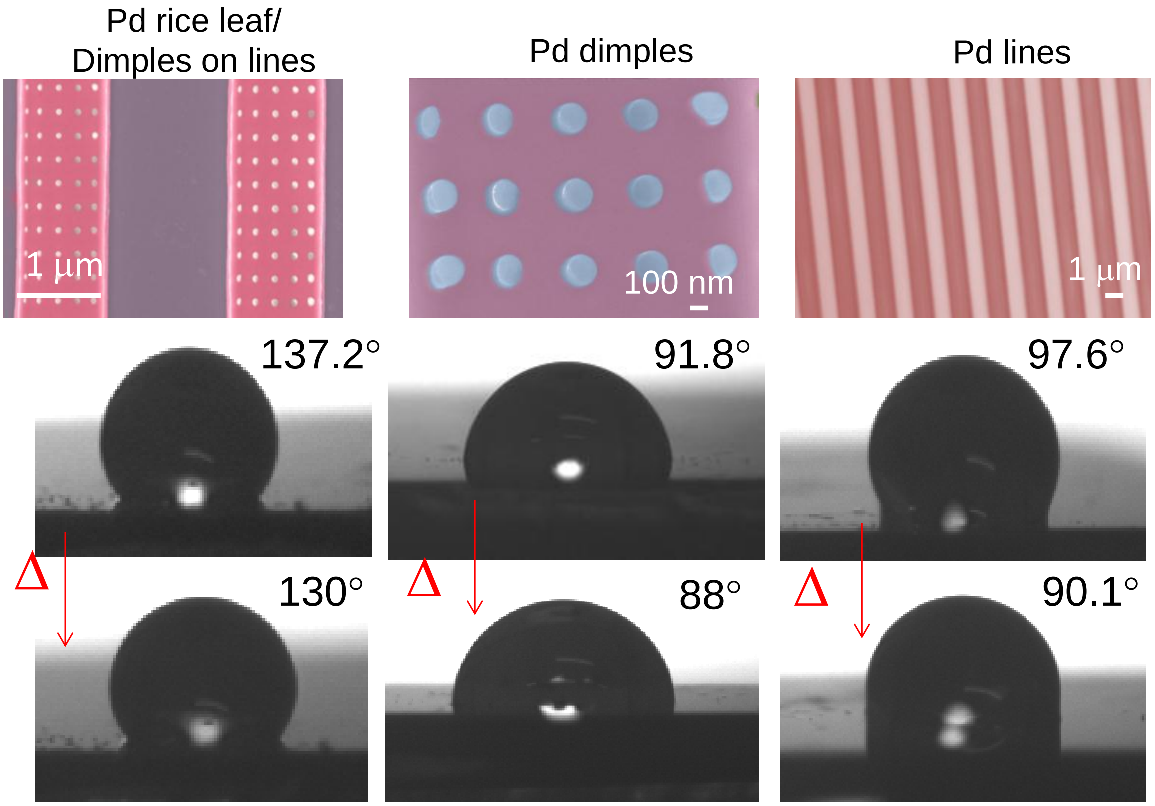

Figure S18. Representative SEM images (false colored) and corresponding contact angles of Pd rice leaf, dimples and lines. The contact angles after heat treatment are also shown below.

<u>S19:</u>

The calculation of enhancement factor, G was done by to Yu et al. (Langmuir, 15 (1998) 16-19),

$$G = (I_{SERS}/I_{norm})(N_{bulk}/N_{surf})$$

where I_{SERS} is the measured SERS intensity for the probe molecules on the Pd nanoparticle surface (2158), I_{norm} is the measured intensity of non-enhanced Raman scattering from the bulk sample (2660), N_{bulk} is the number of the probe molecules under laser illumination in the bulk sample, and Nsurf is the number of the molecules probed on the surface. I_{SERS} and I_{norm} were measured at 652 cm^{-1} (ν(C-S)$_{trans}$ stretch). For bulk, the sampling volume is the product of the area of the laser spot (~ diameter = 1 μm) and the penetration depth (~100 μm). Thus N_{bulk} is

$N_{bulk} = Ah\rho/m$

where A, h, ρ, and m are the area of the laser spot, the penetration depth, the density (0.84 g cm^{-3}), and the molecular weight of hexadecanethiol (258.5 g mol^{-1}), respectively. The N_{surf} can be calculated as follows:

$N_{surf} = CA$

C and A are the surface density of hexadecanethiol (~4.4×10^{14} molecules cm^{-2}) [2, 3] and area of the laser spot on the sample, respectively.

By substituting the values, an enhancement factor of 0.36×10^5 was obtained.

[2] M.D. Malinsky, K.L. Kelly, G.C. Schatz, R.P. Van Duyne, Chain Length Dependence and Sensing Capabilities of the Localized Surface Plasmon Resonance of Silver Nanoparticles Chemically Modified with Alkanethiol Self-Assembled Monolayers, Journal of the American Chemical Society, 123 (2001) 1471-1482.

[3] J. Li, K.S. Liang, N. Camillone Iii, T.Y.B. Leung, G. Scoles, The structure of n-octadecane thiol monolayers self-assembled on Au(001) studied by synchrotron x-ray and helium atom diffraction, The Journal of Chemical Physics, 102 (1995) 5012-5028.
